# Phenylethanoid Glycoside-Enriched Extract Prepared from *Clerodendrum chinense* Leaf Inhibits A549 Lung Cancer Cell Migration and Apoptosis Induction through Enhancing ROS Production

**DOI:** 10.3390/antiox12020461

**Published:** 2023-02-11

**Authors:** Chuda Chittasupho, Sirivan Athikomkulchai, Weerasak Samee, Mingkwan Na Takuathung, Wipawadee Yooin, Kasirawat Sawangrat, Chalermpong Saenjum

**Affiliations:** 1 Department of Pharmaceutical Sciences, Faculty of Pharmacy, Chiang Mai University, Mueang, Chiang Mai 50200, Thailand; 2 Department of Pharmacognosy, Faculty of Pharmacy, Srinakharinwirot University, Ongkharak, Nakhon Nayok 26120, Thailand; 3 Department of Pharmaceutical Chemistry, Faculty of Pharmacy, Srinakharinwirot University, Ongkharak, Nakhon Nayok 26120, Thailand; 4 Department of Pharmacology, Faculty of Medicine, Chiang Mai University, Chiang Mai 50200, Thailand; 5 Clinical Research Center for Food and Herbal Product Trials and Development (CR-FAH), Faculty of Medicine, Chiang Mai University, Chiang Mai 50200, Thailand; 6 Center of Excellence for Innovation in Analytical Science and Technology for Biodiversity-Based Economic and Society (I-ANALY-S-T_B.BES-CMU), Chiang Mai University, Chiang Mai 50200, Thailand

**Keywords:** *Clerodendrum chinense*, leaves, A549 cells, lung cancer, migration, ROS

## Abstract

This study aims to investigate the antioxidant and anti-cancer activities of *Clerodendrum chinense* leaf ethanolic extract. The phenylethanoid glycoside-enriched extract, namely verbascoside and isoverbascoside, was determined in the ethanolic *C. chinense* leaf extract using the validated HPLC method. The ethanolic extract showed DPPH and ABTS free radical scavenging activities with the IC_50_ values of 334.2 ± 45.48 μg/mL and 1012.77 ± 61.86 µg/mL, respectively, and a FRAP value of 88.73 ± 4.59 to 2480.81 ± 0.00 µM. *C. chinense* leaf extract exhibited anti-proliferative activity against A549 lung cancer cells in a dose- and time-dependent manner, with the IC_50_ value of 340.63 ± 89.43, 210.60 ± 81.74, and 107.08 ± 28.90 µg/mL after treatment for 24, 48, and 72 h, respectively. The IC_50_ values of verbascoside, isoverbascoside, and hispidulin were 248.40 ± 15.82, 393.10 ± 15.27, and 3.86 ± 0.87 µg/mL, respectively, indicating that the anti-proliferative effects of the *C. chinense* leaf extract mainly resulted from hispidulin and verbascoside. The selectivity index (SI) of *C. chinense* leaf extract against A549 lung cancer cells vs. normal keratinocytes were 2.4 and 2.8 after incubation for 24 and 48 h, respectively, suggesting the cytotoxic selectivity of the extract toward the cancer cell line. Additionally, the *C. chinense* leaf extract at 250 µg/mL induced late apoptotic cells up to 21.67% with enhancing reactive oxygen species (ROS) induction. Furthermore, the lung cancer cell colony formation was significantly inhibited after being treated with *C. chinense* leaf extract in a dose-dependent manner. The *C. chinense* leaf extract at 250 µg/mL has also shown to significantly inhibit cancer cell migration compared with the untreated group. The obtained results provide evidence of the anti-lung cancer potentials of the *C. chinense* leaf ethanolic extract.

## 1. Introduction

Lung cancer is the leading cause of deaths caused by cancers in the world [1]. In 2022, approximately 236,740 patients are estimated to be diagnosed with lung cancer in the United States [2]. Although lung cancer can be treated with surgery, chemotherapy, radiation therapy, targeted therapy, or a combination of these treatments, the lung cancer five-year survival rate (17.4 percent) is still lower than many other leading cancers [3]. Various lung cancer survivors have a diminished pulmonary function that can be exacerbated by surgery and/or radiation and may be a contraindication to the treatment [2]. It has been known that reactive oxygen species (ROS) plays a dual role in the induction, development, suppression, and treatment of cancers [4]. Excess ROS has been shown to induce nuclear DNA damage, leading to cancer initiation. Interestingly, recent studies have demonstrated a role of ROS in cancer treatment and prevention [5]. In the early stage, high levels of ROS are crucial in promoting tumor initiation and tumor progression [6]. However, it was reported that the massive accumulation of ROS can inhibit tumor growth via two different pathways. The extensive accumulation of ROS can inhibit the tumor cell proliferation signaling pathway and trigger programmed cell death, the cell cycle, and ATP synthesis causing cancer cell death [5,7]. The anti-cancer effects of ROS can be provided by inducing cancer cell death via the activation of apoptotic pathways and the ferroptosis pathway. Pei et al. have shown that ROS suppressed the PI3K/Akt/NF-κB signaling pathway, leading to the inhibition of the proliferation of human A549 cells [8]. Thus, increasing the levels of intracellular ROS in cancer cells is of great interest for lung cancer therapy.

Natural products are pharmacological sources of secondary active metabolites including phenolic compounds, flavonoids, alkaloids, terpenoids, steroids, and tannins. *Clerodendrum chinense* (Osbeck) Mabberley is an evergreen shrub growing up to 2 m tall, in the genus Clerodendrum (Family: Lamiaceae). It is not only a flowering plant grown for ornamental purposes, but it is also cultivated for medical uses. Phenylethanoid glycoside (verbascoside, isoverbascoside, and decaffeoylverbascoside), hispidulin, lupeol, anicariside B5, cornoside, and rengyolone were isolated from the leaves of *C. chinense* [9]. Gao et al. isolated and identified bioactive compounds including beta-sitosterol, clerosterol, daucosterol, caffeic acid, kaempferol, 5,4′-dihydroxy-kaempferol-7-O-beta-rutinoside, acteoside, and leucoseceptoside A in *C. chinense* leaf extract [10]. Root and leaf extracts of *C. chinense* have shown significant analgesic, anti-inflammatory, and antipyretic effects [9,11,12]. In other studies, it was used to treat fever, jaundice, typhoid, and syphilis. The chloroformic flower extract of *C. chinense* was active against *Plasmodium falciparum* with an IC_50_ value of less than 10 µg/mL. The chloroformic stem and flower extracts of *C. chinense* were toxic to *Trypanosoma cruzi* with IC_50_ values of 1.21 and 1.12 µg/mL, respectively. Moreover, the chloroformic extract of the *C. chinense* leaf showed anti-parasitic activities against *T. cruzi* (IC_50_ = 3.39 µg/mL) without an exerted cytotoxic effect on a human cell line [13]. The medicinal use of this plant has been valuable for further investigation.

In this study, the leaves of *C. chinense* were extracted using 95% ethanol as a solvent. The antioxidant activities of *C. chinense* leaf ethanolic extract were performed. The anti-proliferative effects including colony formation and migration inhibitory activities of the extract on human lung cancer cells and their underlying mechanisms related to apoptosis and ROS induction have been reported in this study. The bioactive compounds in *C. chinense* leaf extract were investigated and their anti-cancer activities were compared with the extract. 

## 2. Materials and Method

### 2.1. Materials

Acetonitrile (HPLC grade), 95% ethanol, 37% hydrochloric acid, sodium hydroxide, sulfuric acid, and dichloromethane were purchased from RCI Labscan, Bangkok, Thailand. Gallic acid, Folin–Ciocalteau phenol reagent, aluminum chloride, sodium acetate trihydrate, ferric chloride solution, ferrous sulfate heptahydrate, and potassium persulfate sodium carbonate were obtained from Loba Chemie, Mumbai, India. A549 (ATCC^®^ CCL-185TM) human lung adenocarcinoma cell line was purchased from the American Type Culture Collections (ATCC; Manassas, VA, USA). HaCaT cell line was purchased from Pacific Science, Co., Ltd. (Bangkok, Thailand). Dulbecco’s Modified Eagle Medium (DMEM), fetal bovine serum, 100 U/mL and 100 µg/mL streptomycin, and trypsin-EDTA were purchased from Invitrogen (Carlsbad, CA, USA). Dragendorff’s reagent, phosphotungstic acid, gallic acid, DPPH (2,2-diphenyl-1-picrylhydrazyl), TPTZ (2,4,6-Tris(2-pyridyl)-s-triazine, ABTS (2,2′-azino-bis(3-ethylbenzothiazoline-6-sulfonic acid)), and sulforhodamine B (SRB) were obtained from Sigma-Aldrich (St. Louis, MO, USA). Annexin V-FITC and PI solution were purchased from BD Biosciences, CA, USA). DCF-DA was purchased from Sigma Merck KGaA (Darmstadt, Germany).

### 2.2. Plant Collection and Identification

The leaves of *C. chinense* were collected from Chiang Mai, Thailand. Plant growth and collection occurred at the GPS coordinates: 18°45′48.5″ N 98°59′43.0″ E. Plants were identified by Assistant Professor Sirivan Athikomkulchai with a voucher specimen number (SIRA003) at the Faculty of Pharmacy, Srinakharinwirot University, Nakhonnayok, Thailand.

### 2.3. Plant Extraction

The leaves of *C. chinense* were washed and dried in a hot air oven at 60 °C for 24 h. Then, the dried sample was ground into a fine powder using an electric grinder. Five grams of the plant material were mixed and macerated in 50 mL of 95% ethanol for 72 h. The resultant menstruum was filtered and collected. The plant powder was repeatedly macerated two more times. The extracted menstruum was separated from the solid residue by filtration, and the solvent was evaporated using a rotary evaporator. The yield (%*w*/*w*) of the extract was calculated using the following equation:

(1) $$\mathrm{Yield}\;(\%)=\frac{\mathrm{Weight}\;\mathrm{of}\;\mathrm{extract}}{\mathrm{Weight}\;\mathrm{of}\;\mathrm{dried}\;\mathrm{powder}}\times 100$$

### 2.4. Phytochemical Analysis of C. chinense Leaf Extract 

The crude extract of the *C. chinense* leaf was washed with hexane to remove chlorophyll before flavonoids, phenolic compounds, tannins, and alkaloids testing. The following methods were previously described by Chiangnoon et al. [14]. Briefly, flavonoids were determined using the Shinoda test, phenolic compounds were determined using a ferric chloride assay, tannins were determined using the mixture of 2% *w*/*v* gelatin solution, saturated lead acetate solution, and 1% ferric chloride solution, alkaloids were determined using either Dragendorff’s reagent or Scheibler’s reagent, and terpenoids were determined using the Salkowski technique.

### 2.5. Determination of Total Phenolics in C. chinense Leaf Extract

The Folin–Ciocalteu reaction was used to determine the total phenolic content of the *C. chinense* leaf extract following the method reported by Tunit et al. [15]. Gallic acid solution (3.9 to 125 µg/mL) or *C. chinense* leaf extract at concentrations varying from 39 to 1250 µg/mL was applied to 96-well plates. A total of 100 µL of 10% *v*/*v* Folin–Ciocalteu phenol reagent was added and mixed with 50 µL of 7.5% *w*/*v* sodium carbonate solution. Then, a microplate reader (Spectramax M3, Thermo Scientific, Waltham, MA, USA) was used to measure the solutions’ absorbance at a wavelength of 765 nm after being incubated at room temperature with light protection for 2 h. The total phenolic contents were calculated from the calibration curve for gallic acid and the data were presented as µg gallic acid equivalents per mL of crude extract and µg/mg gallic acid equivalents of dried crude extract.

### 2.6. Determination of Total Flavonoids in C. chinense Leaf Extract

The aluminum chloride colorimetric method was used to examine the total flavonoid levels in *C. chinense* leaf extract [15]. Quercetin solution (3.9–1000 µg/mL) or *C. chinense* leaf extract (78–5000 µg/mL; 100 µL/well) were added to 96-well plates. A total of 30 µL of 5% *w*/*v* sodium nitrate was added and incubated for 5 min. Then, 50 µL of a 2% *w*/*v* aluminum chloride solution was added and incubated for 6 min before being incubated for another 10 min with 1 N sodium hydroxide. The absorbance was measured at 510 nm. The data were calculated and presented as g quercetin equivalents per mL of crude extract and g/mg quercetin equivalents of dried crude extract.

### 2.7. Method Validation of HPLC Analysis for the Identification and Quantification Bioactive Compounds in C. chinense Leaf Extract

The International Conference on Harmonization (ICH, 1996/2005) and the acceptable range according to AOAC 2002 guidelines were used to determine the linearity, precision, accuracy, limit of detection (LOD), limit of quantitation (LOQ), and acceptable range of the HPLC method for the identification and quantification of verbascoside, isoverbascoside, and hispidulin in *C. chinense* leaf extract.

#### 2.7.1. Linearity

The linearity was determined by using the working standard solutions of verbascoside and isoverbascoside at concentrations of 1, 3, 5, 7, and 9 µg/mL and hispidulin at concentrations of 0.3, 0.6, 0.9, 1.2, and 1.5 µg/mL. Each concentration was tested in triplicates. The calibration curves were plotted by the peak area of each standard compound vs. its concentration.

#### 2.7.2. Precision

The interday and intraday precision were identified by quantifying verbascoside standard solution at concentrations of 1.5, 4.8, and 8 µg/mL, isoverbascoside standard solution at concentrations of 2, 5, and 8 µg/mL, and hispidulin standard solution at concentrations of 0.5, 0.9, and 1.3 µg/mL. The interday precision was determined from the standard solution analysis for three consecutive days using the proposed HPLC method. The precision was expressed as a percent relative standard deviation (%RSD).

#### 2.7.3. Accuracy

The accuracy of HPLC method was determined by evaluating recovery percentage. Verbascoside standard solution at concentrations of 1.5, 4.8, and 8 µg/mL, isoverbascoside standard solution at concentrations of 2, 5, and 8 µg/mL, and hispidulin standard solution at concentrations of 0.5, 0.9, and 1.3 µg/mL were analyzed. The recovery percentage was calculated by the following equation:

(2) $$\mathrm{Recovery}\;\left(\%\right)=\frac{\mathrm{Amount}\;\mathrm{found-Amount}\;\mathrm{added}}{\mathrm{Amount}\;\mathrm{added}}\;\times \;100$$

#### 2.7.4. Limit of Detection (LOD) and Limit of Quantitation (LOQ)

LOD and LOQ of the proposed HPLC method were calculated from three calibration curves by the following equations:

(3) $$\mathrm{LOD}=\frac{3\mathrm{SD}\;\mathrm{of}\;\mathrm{intercepts}}{\mathrm{Mean}\;\mathrm{of}\;\mathrm{slope}}$$

(4) $$\mathrm{LOQ}=\;\frac{10\mathrm{SD}\;\mathrm{of}\;\mathrm{intercepts}\;}{\mathrm{Mean}\;\mathrm{of}\;\mathrm{slope}}$$

#### 2.7.5. Identification and Quantification Bioactive Compounds in *C. chinense* Leaf Extract 

Verbascoside, isoverbascoside, and hispidulin were identified and quantified in the *C. chinense* leaf extract using the validated HPLC method using RP-C18 column (ACE 5 C18-AR, 250 × 4.6 mm, 5 μm). The mobile phase consisted of acetonitrile (A) and 0.085% phosphoric acid in water (B) in a gradient elution mode. The gradient elution conditions were 5% of eluent A with a linear gradient to 40% from 0.0 to 20.0 min, followed by a linear gradient to 80% of eluent A at 30.0 min, and this proportion being maintained for 5.0 min with the flow rate of 1.0 mL/min and an injection volume of 50 μL. The solvents were degassed using vacuum. The HPLC samples were filtered through a 0.45 μm membrane filter. The peaks were monitored at 326 nm using a UV–visible detector (YL9120 UV/VIS). The peaks were identified based on the retention time with the comparison of the retention times of standard verbascoside, isoverbascoside, and hispidulin. Their contents in the extracts were quantified by using calibration curves plotted between the peak area and standard concentration.

### 2.8. Antioxidant Assay

#### 2.8.1. 2,2-Diphenyl-1-picrylhydrazyl (DPPH) Radical Scavenging Activity Assay

The DPPH radical scavenging assay was performed using a method described by Chiangnoon et al. [16]. Initially, DPPH was dissolved in absolute ethanol and the extract was diluted in the concentrations ranging from 78 to 2500 µg/mL. A total of 100 µL of various concentrations of gallic acid or *C. chinense* leaf extract were mixed with a 100 µL solution of DPPH. Gallic acid was used as positive control [15]. The mixture was incubated at room temperature with light protection for 30 min. Then, the absorbance was measured at 517 nm. The linear regression analysis was used to calculate the concentration of tested samples that scavenge 50% of the DPPH radical (IC_50_).

#### 2.8.2. 2,2′-Azino-bis (3-Ethylbenzthiazoline-6-sulphonic Acid (ABTS) Radical Scavenging Activity Assay 

ABTS free radical scavenging assay was performed using a method described by Chiangnoon et al. [16]. Initially, ABTS was dissolved with absolute ethanol to a concentration of 7 mM. The ABTS radical cation (ABTS^•+^) was produced by reacting ABTS stock solution with 2.45 mM potassium persulfate in a dark at room temperature for 24 h before use. The ABTS^•+^ solution was diluted with absolute ethanol to achieve a 734 nm absorbance of 0.700 ± 0.02. The reaction mixture composed of various concentrations of gallic acid, a positive control [15], and *C. chinense* leaf extract (180 µL), followed by 20 µL of ABTS^•+^ solution. The absorbance of each reaction mixture was measured after 15 min of incubation time at room temperature in the dark. The linear regression analysis was used to calculate the concentration of tested samples that scavenge 50% of the DPPH radical (IC50).

#### 2.8.3. Ferric Reducing Antioxidant Power (FRAP) Assay

FRAP assay was performed using a method described by Chiangnoon et al. [14]. The FRAP reagent was composed of 10 mM TPTZ in 40 mM HCl, 20 mM FeCl_3_6H_2_O, and acetate buffer (300 mM, pH 3.6) in a 10:1:1 (*v*/*v*) ratio. FRAP reagent (180 µL) was thoroughly mixed with 20 µL of *C. chinense* leaf extract or a positive control, gallic acid. After 30 min of incubation at 37 °C, the absorbance at 595 nm was measured. The standard curve was built with a ferrous sulfate solution (9.8–2500 µM), and the results were expressed in µmol Fe (II) equivalents.

### 2.9. Determination of Cytotoxicity of C. chinense Leaf Extract and Bioactive Compounds Using Sulforhodamine B (SRB) Assay

The A459 cancer cells and HaCaT cells were grown in a DMEM medium supplemented with 10% fetal bovine serum, 100 U/mL penicillin G, 100 g/mL streptomycin at 37 °C, and 5% CO_2_. Every three days, the DMEM media was replaced, trypsinized with 0.25% trypsin-EDTA, and sub-cultured in the same medium. 

Briefly, A549 cells were plated in a 96-well plate for 24 h. After exposure to *C. chinense* leaf extract in the concentrations of 0, 25, 50, 100, 250, 500, and 1000 µg/mL for 24, 48, and 72 h, or verbascoside and isoverbascoside in the concentrations of 12.5, 25, 50, 100, 200, 400, 600, 800, and 1000 µg/mL, and hispidulin in the concentrations of 12.5, 25, 50, 100, 200 µg/mL for 24 h, the cultured cells were fixed for 1 h at 4 °C in ice-cold 10% trichloroacetic acid (TCA) and stained for 30 min at room temperature in 0.4% SRB. Excess dye was washed away with 1% acetic acid several times, and a protein-bound dye was dissolved in 200 μL of 10 mM Tris base solution for determining absorbance using a microplate reader with a 540 nm filter wavelength. The percentage of cell viability was calculated according to the following equation, where the control was the untreated cells:

(5) $$\mathrm{Cell}\;\mathrm{viability}\;(\%)=\frac{A540\;\mathrm{sample}}{A540\;\mathrm{control}}\times 100$$

The effect of *C. chinense* leaf extract on the proliferation of HaCaT keratinocyte cells was determined to observe selective responses of normal cells compared with cancer cells. Briefly, HaCaT cells were plated in a 96-well plate for 24 h. After exposure to *C. chinense* leaf extract in the concentrations of 0, 25, 50, 100, 250, 500, and 1000 µg/mL for 24, 48, and 72 h, the cells were tested for anti-proliferative effects by the method described previously.

### 2.10. Apoptosis Assay

To determine the apoptosis, A459 lung cancer cells (2.5 × 10^5^ cells/well) were plated into 6-well plates for 24 h and exposed to various concentrations of *C. chinense* leaf extract in the concentrations of 0, 50, 100, and 250 µg/mL for 24 h. Trypsin was used to harvest the cells, which were then washed with PBS buffer and resuspended in a 1× apoptosis binding assay buffer. Annexin V-FITC and PI solution were used to identify the apoptotic cells, which were incubated at room temperature for 15 min. Flow cytometry (BD Biosciences, CA, USA) was used to detect cell apoptosis. The results were analyzed by using BD Accuri C6 Plus software.

### 2.11. ROS Formation Assay

To investigate the ROS formation, A549 cells were seeded at a concentration of 2 × 10^5^ cells/well into 6-well plates and were incubated with *C. chinense* leaf extract in the concentrations of 0, 50, 100, and 250 µg/mL for 24 h. Then, cells were trypsinized. The new complete DMEM culture medium with 25 µM 2′,7′-dichlorofluorescin diacetate (DCF-DA) was added and incubated with the cells for 30 min at 37 °C in the dark. ROS generation was determined by flow cytometry (BD Biosciences, CA, USA). Data were analyzed by using BD Accuri C6 Plus software and fluorescent signals were displayed as histograms.

### 2.12. Colony Formation Assay

The colony forming or clonogenic assay is an in vitro quantitative technique used to test a single cell ability to proliferate into a large colony via clonal expansion. A549 cells were cultured with extract at various concentrations (0–250 µg/mL) for 24 h before being switched to the DMEM medium and cultured for another 15 days. After staining the cells with 0.5% crystal violet, the cells were washed with deionized water, air dried, and photographed. A colony was defined as having at least 50 cells per colony. The number of cells was counted and compared to a control group.

### 2.13. Cell Migration Assay

The *C. chinense* leaf extract was tested for its effect on A549 cell migration. In 6-well plates, a monolayer of A549 cells were scratched with a sterile 0.2 mL pipette tip. At 0 h, the cells were washed, and the denuded area was photographed using inverted microscopy (40× magnification). The cells were then treated with different concentrations of *C. chinense* leaf extract (0, 50, 100, and 250 µg/mL) for 48 h. Inverted microscopy was used to capture the images of the control and treatment groups 48 h later. The migration process was represented by the closure of a scratched wound, determined by the denuded area along the scratch. By dividing the area by the length of the scratch, the wound distance was calculated. The results were compared between the control and treatment groups:

(6) $$\mathrm{Relative}\;\mathrm{closure}\;\mathrm{of}\;\mathrm{the}\;\mathrm{scratch}\;(\%)=\frac{\mathrm{Distance}\;\mathrm{at}\;0\;\mathrm{h-Distance}\;\mathrm{at}\;48\;h}{\mathrm{Distance}\;\mathrm{at}\;0\;h}\times 100$$

### 2.14. Statistical Analysis

The data were statistically analyzed using a one-way ANOVA, followed by Tukey’s multiple comparison test as a post-hoc test to determine the significance of the differences (GraphPad Prism 7.02, La Jolla, CA, USA). In all cases, values of *p* < 0.05 were considered statistically significant.

## 3. Results 

### 3.1. Yield of C. chinense Leaf Ethanolic Extract

The *C. chinense* leaf extract was concentrated and dried under a reduced pressure using a rotary evaporator after maceration with 95% ethanol. After weighing the dried extract, the yield was calculated to be 11.20 ± 0.84% *w*/*w*. Extraction is a primary process for the isolation of a variety of bioactive secondary metabolites from plant materials. In this study, ethanol was used for maceration to extract the polar compounds from *C. chinense* leaf. Ethanol was eco-friendly, low toxic, and polar compared with other organic solvents, such as benzene, hexane, and chloroform [16]. Sapiun et al. reported the yield obtained from the extraction of *C. chinense* leaf was equal to 7.6% [17]. The higher yield of extraction observed in this study might be due to the difference in the ratio between the plant and solvent. 

### 3.2. Phytochemical Screening of C. chinense Leaf Ethanol Extract

A qualitative phytochemical analysis of *C. chinense* leaf ethanol extract showed that the extract contained high amounts of phenolic compounds and tannins, followed by flavonoids, alkaloids, and terpenoids (Supplementary Table S1). These results were confirmed by previous reports showing that the ethanol extract of *C. chinense* leaves contained triterpenoids, flavonoids, alkaloids, saponins, tannins, and quinones, except steroids [18]. 

### 3.3. Total Phenolic and Flavonoid Contents of C. chinense Leaf Extract

The total phenolic content of the *C. chinense* leaf extract was determined using Folin–Ciocalteu method and using the gallic acid standard curve equation i.e., y = 0.0151x + 0.0829, r = 0.9993. The total content of flavonoids in *C. chinense* leaf extract was quantified by an aluminum chloride colorimetric assay. The quercetin standard curve equation was y = 0.0013x + 0.0396, r = 0.9962. The total phenolic contents and total flavonoid contents of *C. chinense* leaf extract were 1029.99 ± 21.29 mg GAE/g extract and 326.37 ± 24.54 mg GAE/g extract, respectively (Table 1). 

### 3.4. HPLC Method Validation

The HPLC method for analyzing the three bioactive compounds showed acceptable validation parameters. The calibration curves were obtained by plotting the peak area vs. the concentration of the standards. The accuracy of the method was proved by the linearity within the range of 1–9 µg/mL for verbascoside and isoverbascoside with correlation coefficients of 0.9999 and 0.9994, respectively. The standard curve of hispidulin showed a linearity range of 0.3–1.5 µg/mL, with a correlation coefficient of 0.9992 (Table 2). The percentage of relative standard deviation (%RSD) of verbascoside ranged from 0.25–0.78% and 0.11–0.78% for intraday and interday precisions, respectively (Table 3). The intraday and interday %RSD of isoverbascoside ranged from 0.20–0.64 and 0.13–0.82%, respectively. The %RSD of hispidulin was in the range of 0.26–0.77% and 0.26–0.77%, for intraday and interday precisions, respectively. The %RSD values were lower than 2.0% indicating the precision of the method. The LOD and LOQ of verbascoside, isoverbascoside, and hispidulin were shown in Table 2. The results suggested that this method could be used to analyze all bioactive compounds in *C. chinense* extract with high sensitivity. The HPLC result agreed with report of Wahba et al. suggesting that methanolic leaf extract of *C. chinense* might contain verbascoside, isoverbascoside, and hispidulin as the phytochemical compounds.

### 3.5. Bioactive Compounds in C. chinense Leaf Extract Determined by HPLC Analysis

**Figure 1:**
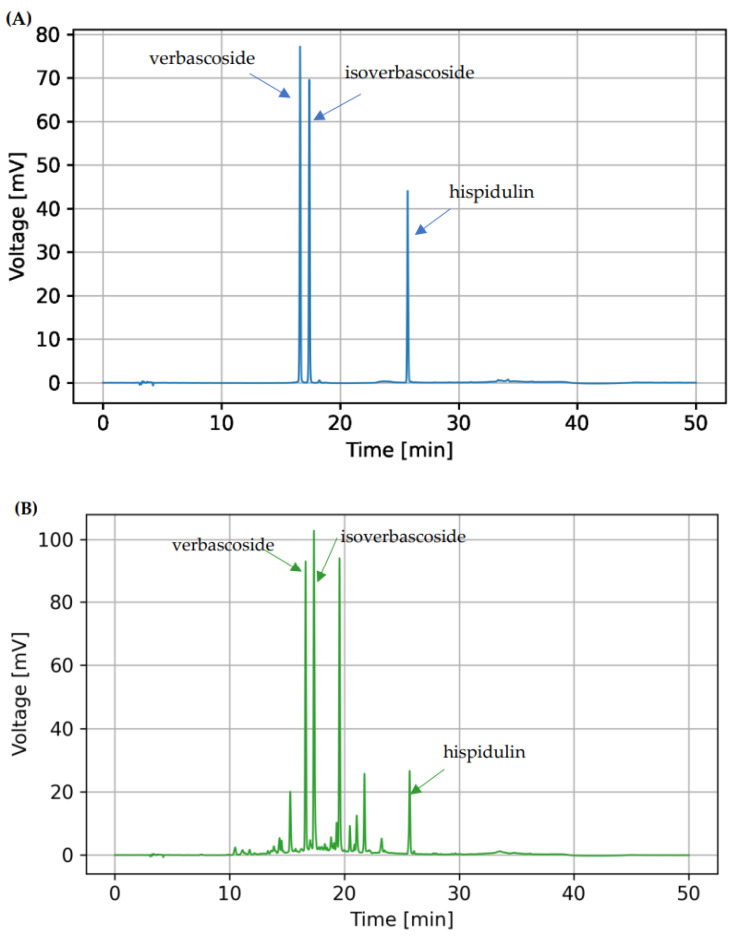
(**A**) HPLC chromatogram of verbascoside (5 µg/mL), isoverbascoside (5 µg/mL), and hispidulin (0.9 µg/mL) standards and (**B**) a HPLC chromatogram of 200 µg/mL *C. chinense* leaf extract.

The identification and quantification of bioactive compounds in *C. chinense* leaf extract are important for the quality control of the extract. In this study, a reversed-phase HPLC was used to identify the phenolic compounds and flavonoids in the leaf extract of *C. chinense*. The identification and quantity of the compounds of *C. chinense* leaf extract by using HPLC was reported here for the first time. The HPLC analysis result indicated that *C. chinense* leaf extract contained verbascoside, isoverbascoside, and hispidulin. The symmetric peaks in the chromatogram of the extract were expressed suggesting the efficient separation of verbascoside, isoverbascoside, and hispidulin from other compounds in the extract, which are shown in Figure 1. The retention times of the verbascoside, isoverbascoside, and hispidulin standards were 16.6, 17.4, and 25.7 min, respectively. The *C. chinense* chromatogram depicted peaks at the retention times of 16.6, 17.4, 19.6, 21.8, and 25.69 min. These results indicated that the *C. chinense* leaf extract was composed of verbascoside, isoverbascoside, and hispidulin. The unidentified peak with a retention time of 19.6 min was thought to be decaffeoylverbascoside. However, the retention time of decaffeoylverbascoside did not correspond with any HPLC peak of *C. chinense* leaf extract (data not shown). Verbascoside and isoverbascoside are caffeoyl phenylethanoid glycosides, classified as phenylpropanoids. Hispidulin (4′,5,7-trihydroxy-6-methoxyflavone) is a flavone derivative. The results suggested that isoverbascoside was the major phytochemical compound present in *C. chinense* ethanolic extract with a respective value of 41.85 ± 0.78 µg/mg extract. Verbascoside was present at 32.37 ± 0.86 µg/mg extract. Hispidulin was contained at 3.20 ± 0.04 µg/mg extract. 

### 3.6. Antioxidant Activities of C. chinense Leaf Extract

**Figure 2:**
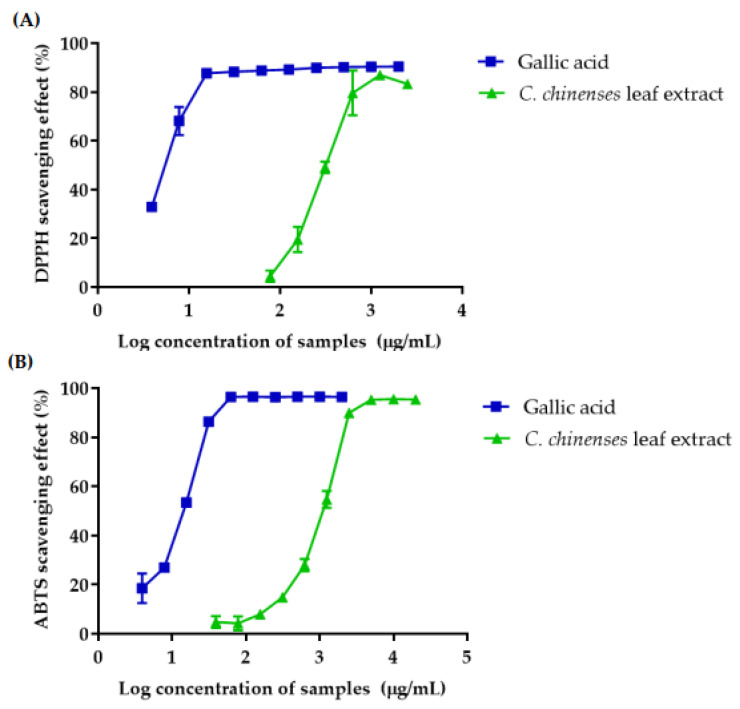

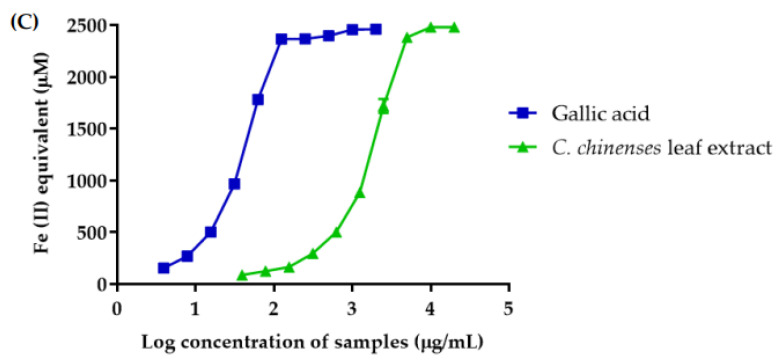
The antioxidant activity of gallic acid and *C. chinense* leaf ethanolic extract determined by (**A**) DPPH free radical scavenging assay; (**B**) ABTS free radical scavenging assay and (**C**) ferric reducing antioxidant power (FRAP) of *C. chinense* leaf extract. Data are shown as the mean ± SD (*n* = 3).

The free radical scavenging activities of the *C. chinense* leaf ethanolic extract was determined using a DPPH radical assay and compared with gallic acid. The results showed that the % DPPH radical scavenging activities of gallic acid and *C. chinense* leaf extract increased with the concentration (Figure 2A). The concentration of gallic acid and *C. chinense* leaf extract that can inhibit 50% of the radical-scavenging effect (IC_50_ values) 5.43 ± 0.11 and 334.2 ± 45.48 µg/mL, respectively.

The free radical scavenging activity of the *C. chinense* leaf extract was confirmed using an ABTS radical assy. Gallic acid and *C. chinense* leaf extract showed ABTS radical scavenging activity in a dose-dependent manner (Figure 2B). The IC_50_ values of gallic acid and *C. chinense* leaf extract were 12.99 ± 0.46 and 1012.77 ± 61.86 µg/mL, respectively. The ABTS assay was more sensitive in identifying antioxidant activity because of the faster reaction kinetics and its response to antioxidant is higher [19].

The ferric-reducing antioxidant powers of gallic acid and *C. chinense* leaf extract are presented in Figure 2C. The calibration curve of ferrous sulfate (Fe^2+^) indicated a good linearity within the range of 9.76–2500 µM (r = 0.9998). The FRAP value was expressed as an Fe^2+^ equivalent. The results showed that the FRAP value of gallic acid and *C. chinense* leaf extract increased with concentration. FRAP values of gallic acid and *C. chinense* leaf extract were in the range of 154.29 ± 1.77 to 2461.44 ± 30.00 µM and 88.73 ± 4.59 to 2480.81 ± 0.00 µM, respectively. These results suggested that the antioxidant activity of *C. chinense* extract involved a ferric-reducing capacity.

### 3.7. Pearson Correlation of Total Phenolic and Flavonoid Contents with Antioxidant Activity of C. chinense Leaf Extract

The total phenolic or flavonoid content of *C. chinense* leaf extract was correlated with its antioxidant activity using Pearson correlation coefficients. The antioxidant activities measured by DPPH and ABTS assays were positively and significantly correlated with total phenolic and flavonoid content (Table 4). Similarly, there was a strong correlation between ferric-reducing potential, as determined by the FRAP assay, and total phenolic and flavonoid content.

### 3.8. Determination of Cytotoxicity of C. chinense Leaf Extract and Bioactive Compounds Using Sulforhodamine B (SRB) Assay

**Figure 3:**
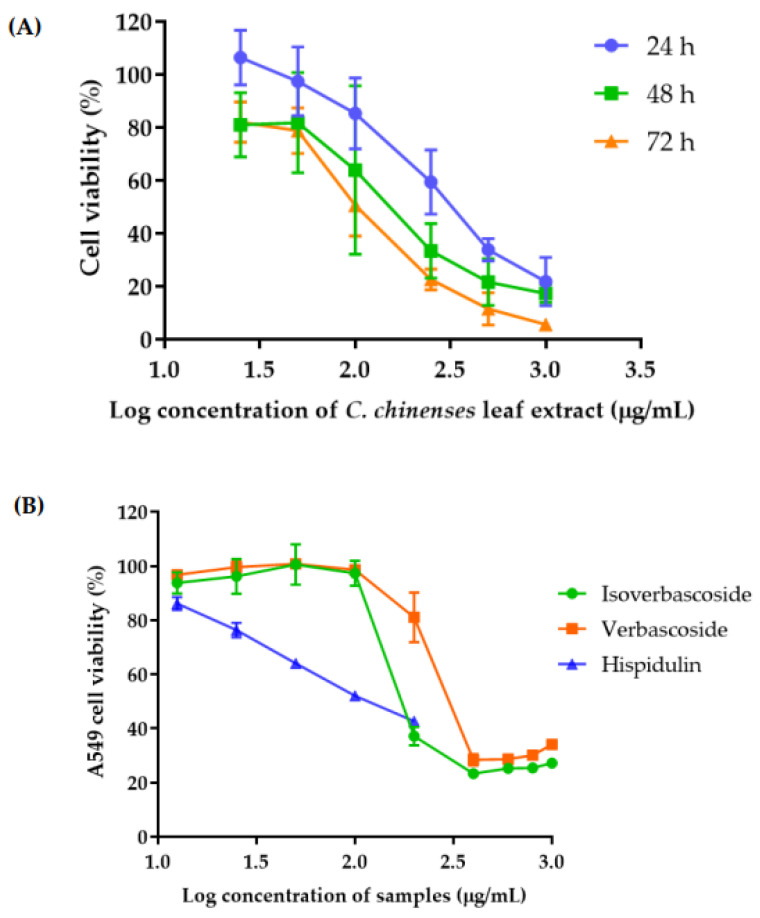
A549 cell viability after incubation with (**A**) *C. chinense leaf* extract for 24, 48, and 72 h and (**B**) verbascoside, isoverbascoside, and hispidulin for 24 h. Data are shown as the mean ± SD (*n* = 3).

The cell proliferation assay was performed to investigate the cell growth inhibitory activity of *C. chinense* leaf extract. The anti-proliferative effect was assessed on the A549 lung cancer cell line 24, 48, and 72 h after treatment with different concentrations of *C. chinense* leaf extract. A dose-dependent decrease in A549 cell viability was observed (Figure 3A). At a concentration of 1 mg/mL of the *C. chinense* leaf extract, the extract reduced cell viability to 21.85 ± 9.10% with the IC_50_ value of 340.63 ± 89.43 µg/mL. The cytotoxicity of *C. chinense* leaf extract against A549 cells was dose and time dependent. At 48 h, the cell viability was reduced to 17.37 ± 3.40% after the treatment of the 1 mg/mL extract. The IC_50_ value of *C. chinense* was 210.60 ± 81.74 µg/mL. After 72 h of treatment of the 1 mg/mL extract, cell viability decreased to 5.63 ± 0.82% with the IC_50_ value of 107.08 ± 28.90 µg/mL. 

The anti-proliferative effects of verbascoside, isoverbascoside, and hispidulin, which were considered as bioactive compounds in *C. chinense* leaf extract were assessed on A549 cell line. A dose-dependent decrease in A549 cell viability was observed (Figure 3B). The IC50 values of verbascoside, isoverbascoside, and hispidulin were 248.40 ± 15.82, 393.10 ± 15.27, and 3.86 ± 0.87 µg/mL, respectively. 

**Figure 4:**
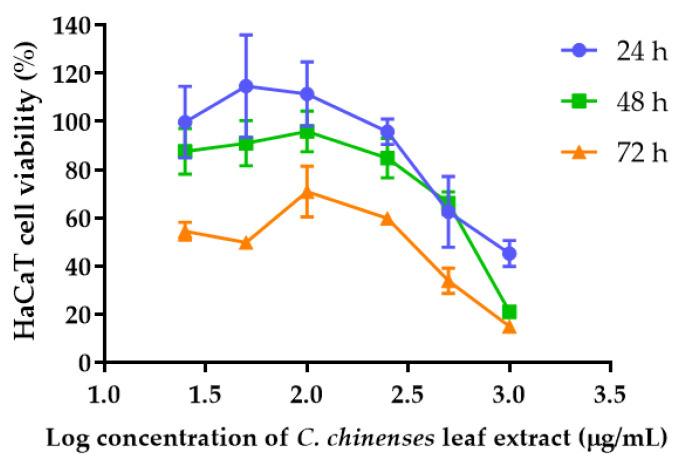
Effect of *C. chinense* leaf extract on HaCaT cell viability after incubation for 24, 48 and 72 h. Data are shown as the mean ± SD (*n* = 3).

The anti-proliferative effect was assessed on the HaCaT keratinocyte cell line 24, 48, and 72 h after treatment with different concentrations of *C. chinense* leaf extract. A dose-dependent decrease in HaCaT cell viability was observed (Figure 4). At a concentration of 1 mg/mL of the *C. chinense* leaf extract, the extract reduced cell viability to 45.25 ± 5.47% with the IC_50_ value of 816.03 ± 159.17 µg/mL. The cytotoxicity of *C. chinense* leaf extract against HaCaT cells was dose and time dependent. At 48 h, the cell viability was reduced to 21.05 ± 2.59% after the treatment of 1 mg/mL extract. The IC_50_ value of *C. chinense* was 585.57 ± 89.44 µg/mL. After 72 h treatment of 1 mg/mL extract, cell viability decreased to 14.98 ± 0.60% with the IC_50_ value of 106.65 ± 26.25 µg/mL. 

To confirm the anti-cancer activity of the *C. chinense* leaf extract, the cytotoxicity of the extract against three more cancer cell lines, including human colorectal adenocarcinoma cell line (Caco-2), human colorectal carcinoma cell line (HCT 116), and human hepatocellular carcinoma cell line (HepG2), were investigated. The results showed that at 200 µg/mL the extract exhibited a cytotoxicity against all the tested cancer cell lines. The IC_50_ values of *C. chinense* leaf extract against Caco-2, HCT 116, and HepG2 cells were 370.95 ± 21.00 µg/mL, 274.45 ± 5.30 µg/mL, and 202.95 ± 1.06 µg/mL, respectively. Data are shown in Supplementary Figure S1. 

### 3.9. Annexin V-FITC/PI for Apoptosis Detection

**Figure 5:**
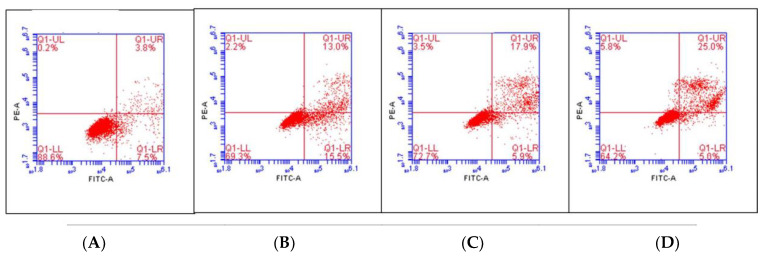
Flow cytometric analysis of early apoptosis, late apoptosis, and necrosis in A549 cells treated with *C. chinense* leaf extract. The dot plot represents the (**A**) untreated A549 cells; (**B**) A549 cells treated with 50 µg/mL *C. chinense* leaf extract; (**C**) A549 cells treated with 100 µg/mL *C. chinense* leaf extract, and (**D**) A549 cells treated with 250 µg/mL *C. chinense* leaf extract.

Apoptosis detection was performed to verify the mechanism of *C. chinense* leaf extract on the growth inhibition of A549 cells. The apoptosis detection was determined in A549 cells treated with *C. chinense* leaf extract at 50, 100, and 250 µg/mL. Figure 5 showed the distribution of A549 cells within four different quadrants (lower right = early apoptosis, upper right = late apoptosis, upper left = necrosis, and lower left = viable cells). The results showed that untreated cells showed a percentage of 83.12 ± 3.02% for viable cells, 3.95 ± 0.14% for late apoptosis, and 0.12 ± 0.07% for necrosis. *C. chinense* at 50, 100, and 250 µg/mL significantly increased in the late apoptotic population to 15.28 ± 1.37%, 14.5 ± 1.25%, and 21.67 ± 1.55%, respectively. An increase in necrotic cells was observed with percentages of 1.97 ± 0.11%, 3.97 ± 0.48%, and 5.75 ± 0.61% upon treatment with *C. chinense* leaf extract at 50, 100, and 250 µg/mL, respectively (Table 5). The percentage of cells undergoing late apoptosis increased significantly, confirming that apoptosis was one of the major mechanisms of cell death induced by *C. chinense* leaf extract.

### 3.10. C. chinense Leaf Extract Induced ROS Generation in A549 Cells

**Figure 6:**
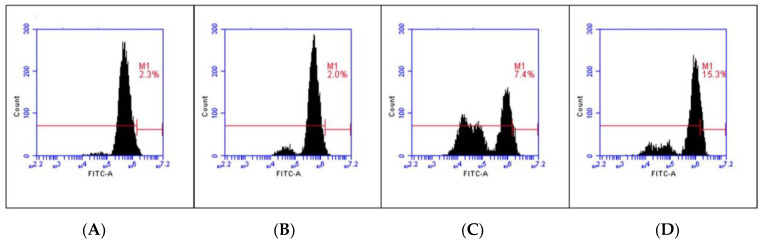
ROS generation in (**A**) untreated A549 cells (**B**) A549 cells treated with 50 µg/mL *C. chinense* leaf extract; (**C**) A549 cells treated with 100 µg/mL *C. chinense* leaf extract; and (**D**) A549 cells treated with 250 µg/mL *C. chinense* leaf extract.

The mechanism underlying the cell apoptosis induction of *C. chinense* leaf extract was investigated. The ROS levels in A549 cells treated with and without *C. chinense* leaf extract at different concentrations were measured using the DCF-DA ROS detection assay kit. The results showed that the intracellular ROS was significantly increased in A549 cells treated with *C. chinense* leaf extract in a concentration-dependent manner (Figure 6), suggesting that *C. chinense* leaf extract-induced ROS production might be a potent ROS-induced apoptosis mechanism in A549 cells. As shown in Table 6, the *C. chinense* leaf extract at 250 µg/mL resulted in 15.95 ± 3.95% ROS formation, compared with control (2.22 ± 0.59%).

### 3.11. C. chinense Leaf Extract Decreased Lung Cancer Cell Colony Formation

**Figure 7:**
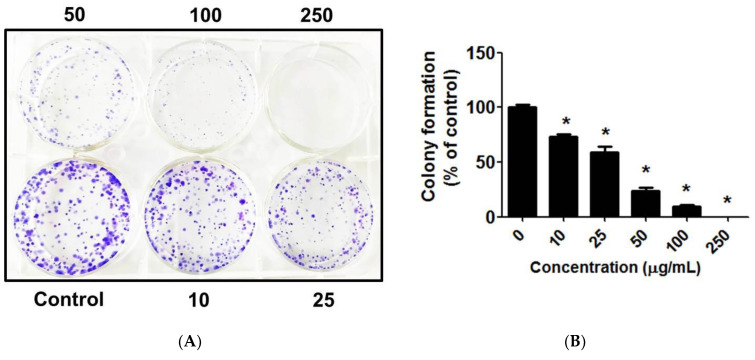
(**A**) Clonogenic formation of A549 cells treated with 0, 10, 25, 50, 100, and 250 µg/mL of *C. chinense* leaf extract, and (**B**) the percentage of colony formations of A549 cells treated with various concentrations of *C. chinense* leaf extract and untreated A549 cells. The data represent the mean ± SD of the three experiments with * *p* < 0.05.

The clonogenic assay measures the capability of a single cell to form a colony. This assay is used to test for the effects of *C. chinense* leaf extract on the growth and proliferative characteristics of A549 cells. The clonogenic formation potential of A549 cells was evaluated as a function of the number of colony formations in the absence or presence of the *C. chinense* leaf extract. The results showed that *C. chinense* leaf extract significantly suppressed the clonogenic formation of A549 cells. The number of colonies of A549 cells significantly decreased after the treatment with *C. chinense* leaf extract in a dose-dependent manner (Figure 7). A549 formed 100 ± 2.24% without treatment. A549 treated with non-cytotoxic concentrations i.e., 10, 20, 50, and 100 µg/mL showed 73.17 ± 2.24%, 59.11 ± 5.26%, 23.96 ± 2.54%, and 9.27 ± 1.78% colony formations, respectively, compared with the control. Cells exposed to 250 µg/mL of the *C. chinense* leaf extract did not form any colonies at all. 

### 3.12. Effect of C. chinense Leaf Extract on Migration of A549 Cells

**Figure 8:**
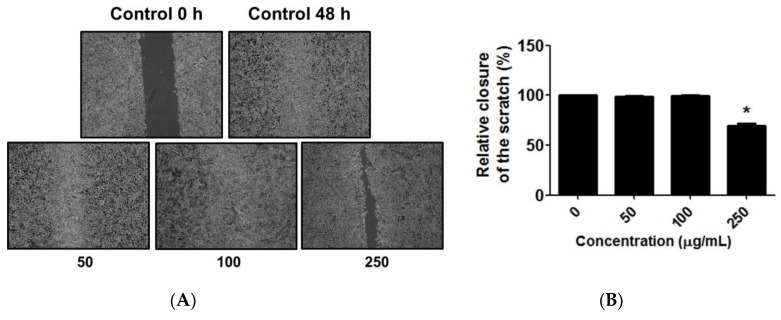
(**A**) Migration of untreated A549 cells and A549 cells treated with 50, 100, and 250 µg/mL *C. chinense* leaf extract, and (**B**) percentage of relative closure of the scratch of untreated A549 cells and A549 cells treated with 50, 100, and 250 µg/mL of *C. chinense* leaf extract. The data represent the mean ± SD of the three independent experiments with * *p* < 0.05.

A cell migration assay was conducted to determine the effects of *C. chinense* leaf extract on the migration capability of A549 cells. The results showed that there was a significant inhibition of A549 cell migration when cells were treated with 250 µg/mL of the *C. chinense* leaf extract compared to the control group. The gap between the control cells was completely covered in 48 h. A549 cells were treated with 0, 50, 100, and 250 µg/mL *C. chinense* leaf extract for 48 h and subjected to a cell migration assay. Quantitative analysis indicated that at 250 µg/mL, the *C. chinense* leaf extract significantly reduced the relative closure of the A549 cells to 69.49 ± 2.05% (Figure 8). 

## 4. Discussion

Phenolic compounds and flavonoids are one of the major classes of phytochemical compounds associated with antioxidant and anti-cancer activity. Phytochemical compounds in plants with the same species can vary from a place of origin, regulated by several factors, such as latitude, longitude, rainfall, temperature, soil quality, habitats, and cultural practices. The polyphenols in the *C. chinense* leaf extract were determined by both the quality and quantity of the chemical content. The total phenolic content in the *C. chinense* leaf extract has never been reported before. However, it was found that the total phenolic content of *C. infortunatum* and *C. grandis* leaf methanolic extracts were found to be 154.54 ± 3.89 mg GAE/g and 24.136 ± 1.81 mg GAE/g extract, respectively [20]. The flavonoid content in the *C. chinense* leaf ethanolic extract was reported to be at least 146 mg quercetin equivalent per g of dry extract. The results revealed that the *C. chinense* leaf extract in this study contained a higher amount of flavonoids compared with the previous study, suggesting that the ratio of extracting solvent and maceration time played a significant role in the bioactive compound extraction [18]. The solvent used for maceration in this study was 95% ethanol, which had a dielectric constant close to that of a previous report (96% ethanol). The maceration process was different in terms of the maceration time and the ratio of plant and extraction solvent. The maceration time was 72 h vs. 24 h and the plant-to-solvent ratio was 1:10 vs. 1:7. Therefore, we concluded that prolonged maceration time and higher amount of solvent resulted in a higher yield and flavonoid concentration of the *C. chinense* leaf extract.

The HPLC analysis results suggested that isoverbascoside was the major phytochemical compound present in the *C. chinense* ethanolic extract. Verbascoside and hispidulin were detected in the extract. Wahba et al. isolated the phytochemical compounds of *C. chinense* leaves extracted by 80% methanol. Three phenylpropanoid glycosides, including verbascoside, isoverbascoside, decaffeoylverbascoside, one flavonoid (hispidulin), two cyclohexylethanoids (cornoside and rengyolone), and icariside B5 were identified by comparing the obtained physicochemical data with the spectra of previous reports [9].

Antioxidants have the ability to reduce free radicals. Phytochemical compounds, such as phenolic compounds and flavonoids, act as antioxidants with redox and metal-chelating properties. The antioxidant activities of the *C. chinense* leaf extract may be attributed to the presence of polyphenolic compounds and flavonoids that act as a free radical scavenger via hydrogen atom donation and a reducing agent. The cell viability assay indicated that the cytotoxicity of the standards was in the following order: hispidulin > verbascoside > isoverbascoside. The cytotoxicity of the *C. chinense* leaf extract was suggested to be mainly due to hispidulin and verbascoside. The selectivity index (SI) of the *C. chinense* leaf extract against A549 lung cancer cells vs. normal keratinocytes was 2.4, 2.8, and 1.0 after incubation for 24, 48, and 72 h, respectively. These results indicated that *C. chinense* was more selectively cytotoxic to lung cancer cells within 24–48 h. 

The anti-cancer efficacy of natural polyphenols has largely been attributed to their potent antioxidant activity, as well as their abilities to modulate molecular targets and the signaling pathways of cancer cell proliferation, differentiation, apoptosis, metastasis, and multidrug resistance [21]. The anti-cancer property of polyphenols involved the scavenging of reactive oxygen species (ROS) and oxidizing agents catalyzing the tumor growth [22]. In addition, polyphenols participated in cellular molecular mechanisms related to cancer occurrence and development. Hence, they may also be used for cancer prevention, not only anti-cancer activity. The anti-cancer activity of *C. chinense* leaf extract macerated in 70% ethanol against A549 lung cancer cells after 24 h-exposure has been reported. However, the IC_50_ values of those reported was higher than our findings (436.91 µg/mL vs. 340.63), suggesting that the extraction solvent played an important role in the anti-cancer activity [23]. 

The role of antioxidants and their ability to act as prooxidants depending on concentration and the nature of surrounding molecules [24,25]. Some antioxidants have been shown to have prooxidant properties. The presence of metal ions, the concentration of the antioxidant in matrix environments, and its redox potential are all factors that can influence an antioxidant’s function and convert it to a prooxidant [24]. Transition metal-containing flavonoids, such as quercetin and kaempferol, have been shown to act as prooxidants [26]. In the presence of the transition metal, these flavonoids caused DNA damage and lipid peroxidation. Phenolic compounds can also be prooxidant, especially in the presence of redox-active metals. The presence of iron or copper catalyzes their redox cycling, which can result in the formation of phenolic radicals, which can cause lipid and DNA damage [27]. As the redox balance of intracellular ROS levels is required for tumor development and progression, stimulating ROS formation is one of strategies for cancer therapy via elevated oxidative stress through various signaling pathways. In addition, the increased level of ROS production has been associated with cancer cell apoptosis and cell cycle arrest. As such, *C. chinense* leaf extract could potentially serve as a chemotherapeutic agent for lung cancer cells.

In addition to evaluating cellular growth and the cytotoxic or genotoxic effects of various agents with potential clinical application, clonogenic assays assess the ability of cells to maintain cell division over time as a result of damage to chromosomes and/or apoptosis. This assay is significant when analyzing therapeutic resistance, since short-term cytotoxicity assays cannot detect drug resistance [24]. The cell migration assay revealed that high doses of *C. chinense* leaf extract led to the significant inhibition of migration of A549 cells, suggesting a low potential of cell migration inhibition of this extract. In addition, this effect might be partly due to the apoptosis induced by *C. chinense* leaf extract.

The anti-cancer mechanism of *C. chinense* leaf extract might be a result of the anti-cancer activities of verbascoside, isoverbascoside, and hispidulin. The major compound in *C. chinense* leaf extract analyzed by HPLC is isoverbascoside (41.85 ± 0.78 µg/mg extract). The most active compound is hispidulin. This statement was supported by the fact that the cytotoxicity of the chemical compounds against A549 lung cancer cells was in the following order: hispidulin > verbascoside > isoverbascoside. Verbascoside has been shown to have anti-cancer activity both in vitro and *in vivo*. Verbascoside inhibited the signaling cascades of Rac-1, Zeb-1 (zinc finger E-box binding homeobox 1), Arp2 (actin-related proteins), Pak1 (p21 (RAC1) activated kinase 1), VEGF (vascular endothelial growth factor), and HIF-1 in colorectal cancer cells, according to Seyfi et al. [25]. This compound increased the invasion and migration of the U87 brain tumor cell line when combined with temozolomide, indicating a synergistic therapeutic capability for the treatment of glioblastoma [26]. Verbascoside had anti-tumor activity comparable to 5-fluorouracil in nude mice with colorectal cancer [27]. It has been shown to sensitize Caco-2 cancer cells to 5-fluorouracil by inhibiting the AKT/PI3K pathway [28]. Vasincu et al. reported that verbascoside increased the rate of apoptosis in A549, MCF-7, and HT-29 cells by 2.3, 2.5, and 7.5-fold, respectively, when compared to control cells by increasing ROS production [29]. Verbascoside inhibited the growth of a human leukemia cell line in a time- and dose-dependent manner, and according to Kyung-Won Lee et al., flow cytometric analysis revealed that verbascoside inhibited cell cycle progression in the G1 phase in HL-60 cells [30]. Verbascoside treatment at high doses caused apoptosis in rat prostate cancer cells. By inhibiting the HMGB1/RAGE pathway, verbascoside was shown to significantly reduce cell proliferation and migration in human prostate tumor cell lines [31]. Verbascoside alone has not been reported to show anti-cancer activity in animal models. However, verbascoside-Ni nanoparticles effectively reduced the volume of the implanted tumors [32]. In addition, the treatment of verbascoside, immobilized on to cadmium telluride quantum dots, effectively inhibited the human hepatoma HepG2/ADM nude mice tumor growth [33]. 

Isoverbascoside has been shown to inhibit MGC 803 gastric cancer cell proliferation by causing G0/G1 arrest and regulating the expression of cell cycle-related proteins [34]. Isoverbascoside has shown great potential in suppressing both in vitro and in vivo ovarian cancer cell growth [35]. The results revealed that OVCAR-3 tumor growth and tumor volume was significantly suppressed by isoverbascoside administration, compared with the control group [35]. Lv et al. demonstrated that hispidulin induced NSCLC xenograft tumor growth inhibition and apoptosis in nude mice [36]. Isoverbascoside markedly inhibit cell proliferation of human gastric cancer cells by suppressing cell tumorigenicity, alkaline phosphatase, and lactate dehydrogenase activities, and causing G0/G1 arrest. It also up-regulated the expression of G1/S checkpoint-related proteins, i.e., p53, p21/WAF1, and p16/INK4, and suppressed the expression of C-myc protein [34]. 

Among the three isolated compounds in *C. chinense* leaf extract, hispidulin is the most promising natural agent to be developed as an anti-cancer drug in the future. Hispidulin showed antiproliferative properties against a variety of human cancers and provoked cancer cell death via multiple mechanisms, including ROS-mediated apoptosis induction, arresting the cell cycle, and targeting multiple signaling pathway of cancer cell progression [37]. However, further preclinical and clinical studies are needed to verify the use of hispidulin as a chemosensitizing agent in clinical settings. Hispidulin has been shown in vitro and in animal studies to have significant anti-cancer activity in a variety of cancer types by inhibiting tumor growth and neovascularization, suppressing tumor cell metastasis, and inducing apoptosis [38]. The anti-cancer activity of hispidulin has been linked to an anti-proliferative effect, cell cycle arrest induction, and mitochondria-mediated apoptosis. Peng et al. discovered that hispidulin inhibited the PI3k/Akt signaling pathway, causing mitochondrial-mediated apoptosis and excessive ROS production. As a result, ROS could be the upstream signaling molecule that mediates hispidulin’s anti-cancer activity [39]. In a dose-dependent manner, hispidulin inhibited the growth of NCI H460 and A549 cells while promoting apoptosis via the increased generation of reactive oxygen species (ROS). Hispidulin-induced apoptosis in NSCLC cells by increasing the expression of caspase 3 and poly [ADP ribose] polymerase. Furthermore, hispidulin activated endoplasmic reticulum stress in NCI H460 cells. [36]. The results suggested that the antioxidant and anti-cancer activities of the *C. chinense* leaf extract might be attributed to the verbascoside, isoverbascoside, and hispidulin enriched in the extract. 

Syntheses of isoverbascoside and hispidulin have been reported. Zhifei et al. applied the regioselective glycosylation, which avoids the selective protection/deprotection of the hydroxyl groups and provides a shorter synthetic approach toward isoverbascoside [40] Kawada et al. performed a total chemical synthesis of isoverbascoside by the deacetylation and caffeoyl migration of verbascoside acetate with benzyl groups, followed by debenzylation by catalytic transfer hydrogenation [41]. The synthetic approaches to hispidulin, a natural flavone, have been developed by several research groups. Kavvadias and coworkers developed a method to synthesize hispidulin from a 2,4,6-trihydroxyacetophenone compound [42]. Chao et al. synthesized hispidulin by a different method and used the synthesized compound to determine the binding mode of hispidulin as a Pim-1 kinase inhibitor [43]. Lin et al. demonstrated a synthetic method for the synthesis of hispidulin [44].

The greater antitumor efficacy of the combination of hispidulin and the anti-cancer drug sunitinib has been reported. Gao et al. demonstrated that the combination of hispidulin and sunitinib inhibited the growth and angiogenesis of xenografts generated from Caki-1 significantly and decreased the expression of proteins promoting xenograft growth and angiogenesis [45]. Another study showed that hispidulin exhibited anti-tumor activity and potentiated the anti-tumor activity of TMZ in glioblastoma by inhibiting cancer cell proliferation and inducing cell apoptosis [46]. Recently, natural compounds, such as curcumin, resveratrol, dihydroartemisinin, and paclitaxel, have been shown to enhance tumor radiotherapy sensitivity by inhibiting tumorigenesis and radioresistance. Hispidulin demonstrated synergistic anti-tumor effects when combined with gemcitabine, 5-fluoroucil, sunitinib, and temozolomide. The combination of hispidulin and chemotherapeutic drugs reduced chemotherapeutic drug efflux, increased cancer cell chemosensitivity, and reversed drug resistance [39].

## 5. Conclusions

*C. chinense* leaf ethanolic extract contained polyphenolic compounds, tannins, flavonoids, alkaloids, and terpenoids. The extract showed antioxidant activities, which are attributed to the presence of phenolic compounds and flavonoids. The polyphenolic compounds of this extract were verbascoside and isoverbascoside which classified as phenylethanoid glycoside, and the flavonoid compound was hispidulin. *C. chinense* leaf extract exhibited anti-lung cancer activity in vitro by reducing lung cancer cell proliferation, inducing cell apoptosis, decreasing colony formation, and reducing cell migration through enhancing ROS production. Thus, *C. chinense* leaf extract may be a promising candidate for the development of chemotherapeutic agents or natural active pharmaceutical ingredients (NAPIs) to prevent and treat lung cancer. 

## Figures and Tables

**Table 1:** Total phenolic and total flavonoid contents of *C. chinense* leaf ethanol extract.

	Total Phenolic Content (mg GAE/g)	Total Flavonoid Content (mg GAE/g)
*C. chinense* leaf ethanol extract	1029.99 ± 21.29 mg	326.37 ± 24.54

All data are mean ± SD of triplicate measurement.

**Table 2:** Method validation parameters by the proposed HPLC method.

Parameters	Verbascoside	Isoverbascoside	Hispidulin
Regression equation	y = 89.026x − 2.301	y = 89.577x − 32.786	y = 318.83x − 21.409
Correlation coefficient (r)	0.9999	0.9999	0.9993
Linear range (µg/mL)	1–9	1–9	0.3–1.5
LOD (µg/mL)	0.0039	0.0790	0.0213
LOQ (µg/mL)	0.0130	0.2633	0.0711

Where y and x denote the peak area and corresponding concentration (µg/mL), respectively.

**Table 3:** Accuracy and precision of the proposed HPLC method. Data are shown as the mean ± SD (*n* = 3).

Concentration	Verbascoside			Isoverbascoside			Hispidulin		
	1.5	4.8	8.0	2.0	5.0	8.0	0.5	0.9	1.3
Mean recovery, %	95.69 ± 0.31	98.04 ± 1.30	102.51 ± 2.17	104.45 ± 1.42	100.90 ± 0.34	103.64 ± 1.36	97.70 ± 0.09	102.56 ± 0.95	103.35 ± 0.40
	Day 1	Day 2	Day 3	Day 1	Day 2	Day 3	Day 1	Day 2	Day 3
Intraday, %RSD	0.25–0.78	0.11–0.46	0.12–0.50	0.20–0.64	0.32–0.38	0.13–0.82	0.26–0.77	0.29–0.68	0.32–0.65
Interday, % RSD	0.33–2.12			0.33–1.36			0.09–0.93		

**Table 4:** Pearson correlation coefficients of total phenolic and flavonoid contents and antioxidant activities of *C. chinense* leaf ethanolic extract measured by DPPH, ABTS, and FRAP assays.

	Total Phenolic Content (Gallic Acid Equivalent)	Total Flavonoid Content (Quercetin Equivalent)
DPPH assay	0.9557 *	0.8829 *
ABTS assay	0.9990 ****	0.9997 ****
FRAP assay	0.9994 ****	0.9987 ****

* Indicated *p* < 0.05 and **** indicated *p* < 0.0001.

**Table 5:** The percentage of viable, late apoptosis, and necrotic cells in treatment with *C. chinense* leaf extract on A549 cells.

Group of Treatment	Viable Cells (%)	Late Apoptosis (%)	Necrosis (%)
Control	83.12 ± 3.02	3.95 ± 0.14	0.12 ± 0.07
CCL 50 µg/mL	75.60 ± 1.18 *	15.28 ± 1.37 *	1.97 ± 0.11
CCL 100 µg/mL	68.10 ± 1.29 *	14.50 ± 1.25 *	3.97 ± 0.48
CCL 250 µg/mL	63.57 ± 3.35 *	21.67 ± 1.55 *	5.75 ± 0.61 *

The results are shown as mean ± SD of three independent experiments with * *p* < 0.05.

**Table 6:** Percentage of ROS formation of A549 cells treated with various concentrations of *C. chinense* leaf extract.

	Control	Extract 50 µg/mL	Extract 100 µg/mL	Extract 250 µg/mL
ROS formation (%)	2.22 ± 0.59	1.70 ± 0.33	5.42 ± 1.96	15.95 ± 3.95 *

The results are shown as mean ± SD of three independent experiments with * *p* < 0.05.

## Data Availability

The data presented in the study are available in this manuscript.

## Supplementary Materials

The following supporting information can be downloaded at: https://www.mdpi.com/article/10.3390/antiox12020461/s1, Table S1: Phytochemical analysis of *C. chinense* leaf ethanol extract, and Figure S1: The viability of (A) Caco-2 (B) HCT 116, and (C) HepG2 cells after incubation with *C. chinense* leaf extract. Data are shown as the mean ± SD (*n* = 3).
