# Supplementary material for: Phenylethanoid Glycoside-Enriched Extract Prepared from Clerodendrum chinense Leaf Inhibits A549 Lung Cancer Cell Migration and Apoptosis Induction through Enhancing ROS Production
Source: Antioxidants (Basel). 2023 Feb 11;12(2):461. doi: 10.3390/antiox12020461 (PMC9952440; doi:10.3390/antiox12020461)
Supplement: Supplementary file 1 [file antioxidants-12-00461-s001.zip › antioxidants-2156644-supplementary.pdf]

Phenylethanoid glycoside-enriched extract prepared from *Clerodendrum chinense* leaf inhibits A549 lung cell migration and apoptosis induction through enhancing ROS production

Chuda Chittasupho[1], Sirivan Athikomkulchai[2], Weerasak Samee[3], Mingkwan Na Takuathung[4], Wipawadee Yooin[1, 5], Kasirawat Sawangrat[1, 5], Chalermpong Saenjum[1, 5*]

[1]Department of Pharmaceutical Sciences, Faculty of Pharmacy, Chiang Mai University, Mueang, Chiang Mai 50200, Thailand

[2]Department of Pharmacognosy, Faculty of Pharmacy, Srinakharinwirot University, Ongkharak, Nahonnayok 26120, Thailand

[3]Department of Pharmaceutical Chemistry, Faculty of Pharmacy, Srinakharinwirot University, Ongkharak, Nahonnayok 26120, Thailand

[4]Department of Pharmacology, Faculty of Medicine, Chiang Mai University 50200, Thailand

[5]Center of Excellence for Innovation in Analytical Science and Technology for Biodiversity-Based Economic and Society (I-ANALY-S-T_B.BES-CMU), Chiang Mai University, Chiang Mai 50200, Thailand

*Corresponding author. E-mail: chalermpong.s@cmu.ac.th

Supplementary Table S1. Phytochemical analysis of *C. chinense* leaf ethanol extract.

Test	Results
Flavonoids	
Shinoda test	++ (Red-orange color)
Phenolic compounds	
Ferric chloride test	+++ (Greenish-blue color)
Tannin	
Gelatin solution	+++ (Precipitation)
Lead acetate saturated solution	+++ (Precipitation)
1% Ferric chloride	+++ (Greenish-black color)
Alkaloids	
Dragendorff's reagent	+ (Orange precipitation)
Scheibler's reagent	+ (White precipitation)
Terpenoids	
Salkoski test	+ (Reddish-brown color ring interface)

+ Presence – Absence

Supplementary data of cytotoxicity study
Cell culture and cytotoxicity assay

The human colorectal adenocarcinoma Caco-2, human colorectal carcinoma HCT 116, hepatocellular carcinoma HepG2 cells were obtained from American Type Culture Collection (Manassas, VA). The cells were cultured in DMEM containing 10%FBS, 100 U/mL penicillin-streptomycin under at 37 °C and 5% CO_2. For the viability assay, the cells ($5x10^3$ cells) were seeded into each well of a 96-well plate and incubated with and without the sample at different concentrations (0–200 µg/mL) for 72 h. Then, the cultured media was removed and the new cultured media containing cell viability reagent namely PrestoBlue was added and incubated at 37 °C for 1 h. The color intensity was measured at 560 and 595 nm using a microplate reader. The cell viability of the tested sample was calculated compared to the negative control.

Results

The cytotoxicity of *C. chinense* leaf extract was investigated in human colorectal adenocarcinoma cell line (Caco-2), human colorectal carcinoma cell line (HCT 116), and human hepatocellular carcinoma cell line (HepG2). The results showed that at 200 µg/mL the extract showed cytotoxicity against all tested cancer cell lines. The IC_{50} values of *C. chinense* leaf extract against Caco-2, HCT 116, and HepG2 cells were 370.95±21.00 µg/mL, 274.45±5.30 µg/mL, and 202.95±1.06 µg/mL, respectively. These results indicated that *C. chinense* leaf extract also exhibited anti-cancer activities against colorectal and liver cancer cells, confirming the anti-cancer activity of *C. chinense* leaf extract.

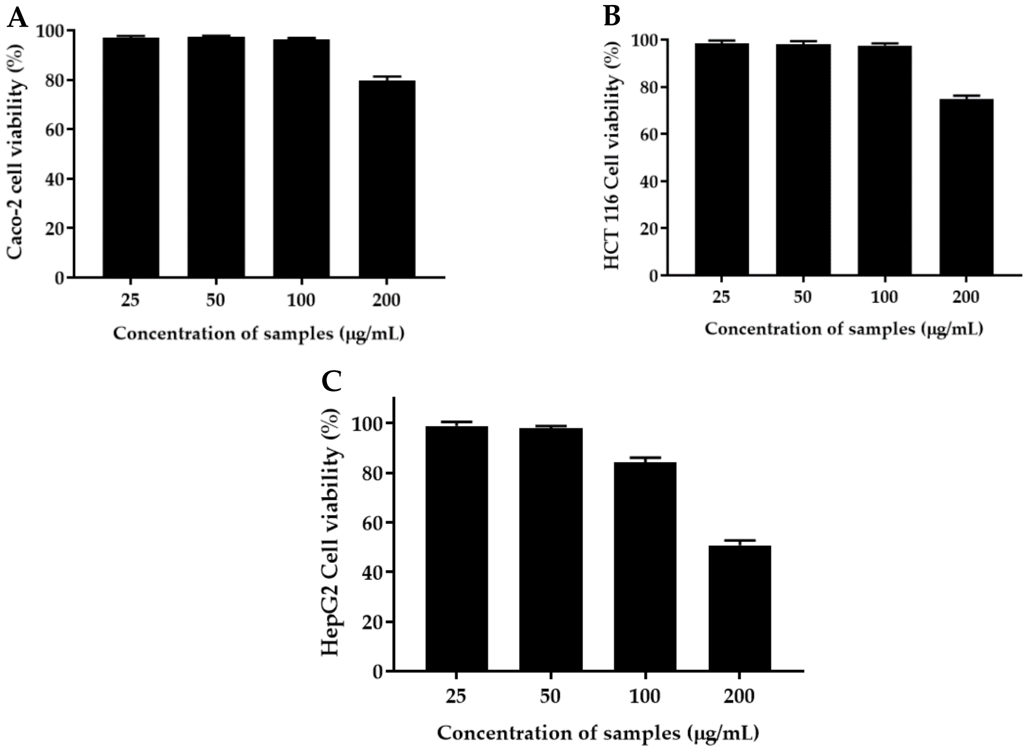

Figure S1 The viability of (A) Caco-2 (B) HCT 116, and (C) HepG2 cells after incubation with *C. chinense* leaf extract. Data are shown as the mean ± SD (n = 3).
